# StatFaRmer: cultivating insights with an advanced R shiny dashboard for digital phenotyping data analysis

**DOI:** 10.3389/fpls.2025.1475057

**Published:** 2025-03-13

**Authors:** Daniil S. Ulyanov, Alana A. Ulyanova, Dmitry Y. Litvinov, Alina A. Kocheshkova, Alexandra Yu. Kroupina, Nadejda M. Syedina, Viktoria S. Voronezhskaya, Andrey V. Vasilyev, Gennady I. Karlov, Mikhail G. Divashuk

**Affiliations:** ^1^ All-Russia Research Institute of Agricultural Biotechnology, Moscow, Russia; ^2^ School of Biological and Medical Physics, Moscow Institute of Physics and Technology, Dolgoprudny, Moscow Region, Russia

**Keywords:** high-throughput plant phenotyping, phenotypic data visualization, time series analysis, digital phenotyping platforms, genotype-phenotype analysis, statistical analysis of phenotypic data, open-source software, automated data analysis

## Abstract

Digital phenotyping is a fast-growing area of hardware and software research and development. Phenotypic studies usually require determining whether there is a difference in some trait between plants with different genotypes or under different conditions. We developed StatFaRmer, a user-friendly tool tailored for analyzing time series of plant phenotypic parameters, ensuring seamless integration with common tasks in phenotypic studies. For maximum versatility across phenotypic methods and platforms, it uses data in the form of a set of spreadsheets (XLSX and CSV files). StatFaRmer is designed to handle measurements that have variation in timestamps between plants and the presence of outliers, which is common in digital phenotyping. Data preparation is automated and well-documented, leading to customizable ANOVA tests that include diagnostics and significance estimation for effects between user-defined groups. Users can download the results from each stage and reproduce their analysis. It was tested and shown to work reliably for large datasets across various experimental designs with a wide range of plants, including bread wheat (*Triticum aestivum*), durum wheat (*Triticum durum*), and triticale (× *Triticosecale*); sugar beet (*Beta vulgaris*), cocklebur (*Xanthium strumarium*) and lettuce (*Lactuca sativa*), corn (*Zea mays*) and sunflower (*Helianthus annuus*), and soybean (*Glycine max*). StatFaRmer is created as an open-source Shiny dashboard, and simple instructions on installation and operation on Windows and Linux are provided.

## 1 Introduction

Digital phenotyping is crucial for tackling challenges like climate change, population growth, and environmental stress (Pieruschka and Schurr, 2019). Until recently, traditional methods of phenotyping did not align with the capabilities of high-throughput genome sequencing and genotyping techniques. These limitations have prompted scientists from diverse fields, including agriculture and engineering, to explore new technologies for phenotyping (Al-Tamimi et al., 2022).

The rapid advancement of high-throughput plant phenotyping (HTPP) tools has resulted in platforms generating enormous amounts of data (Li et al., 2021). High-throughput experiments are conducted both in controlled and field settings through the extensive use of frequent, non-destructive automatic sampling and/or monitoring of several hundreds to thousands of plants within a short period (Al-Tamimi et al., 2022). Comprehensive phenome-wide data facilitate comparisons across populations, enabling phenomics to characterize diverse traits, including structural, physiological, and performance metrics under different environmental conditions (Rahaman et al., 2015; Demidchik et al., 2020). When dealing with large volumes of data, the statistical power of analysis increases. This is particularly true in cases where time series data are involved. Also, in HTPP input data can be highly heterogeneous, such as during studies of different plant varieties and genotypes and different treatments and sites of the studies. Analysis and interpretation of data by appropriate techniques and tools are required. To maximize the potential of HTPP, it is essential for researchers to be able to manage large datasets. This necessitates the efficient collection and management of data, which is most effectively achieved through automated processes (Araus et al., 2022).

A number of companies and research institutes have developed high-capacity phenotyping platforms, both indoor and outdoor, such as Traitmill (CropDesign, Belgium) (Lobet, 2017), HyperAIxpert (LemnaTec, Germany) (HyperAIxpert Family - LemnaTec), and The Plant Accelerator (Australian Plant Phenomics Network, Australia) (Australian Plant Phenomics Network, [[NoYear]]). Such platforms are used to assess crop features related to productivity and tolerance to stressors like salinity (Lazarević et al., 2021; Li et al., 2022), drought (Hein et al., 2021; Joshi et al., 2021; Kim et al., 2021; Javornik et al., 2023), low temperature (Islam et al., 2021). They employ advanced technologies, including imaging stations, automated systems, and proprietary software, to conduct efficient analysis of plant characteristics. In addition, various software solutions have been developed to automatically extract standard features from images, such as plant height and width, utilizing open-source platforms such as HTPheno (Hartmann et al., 2011) and the Integrated Analysis Platform (IAP) (Yang et al., 2020).

Regardless of the method used to collect data on plant phenotype, the next essential step is statistical analysis of the results. Critical for the average user is the self-sufficiency in employing analytical tools, eliminating the need for recurrent solution development. Regrettably, the embedded analytical tools in mainstream digital phenotyping platforms frequently fall short in managing extensive datasets with diverse attributes.

Contemporary methodologies of time series analyses of phenotypic data prevalent in recent publications routinely entail procedures such as outlier identification, percentage transformations, ANOVA, and *post-hoc* Tukey’s test (Kjaer and Ottosen, 2015; Minervini et al., 2017; Parmley et al., 2019; Kim et al., 2020; Leiva et al., 2021; Nyonje et al., 2021; Schmidt et al., 2023; Tripodi et al., 2024). With the rapid increase in data volume, it has become clear how crucial it is to be able to seamlessly visualize and validate digital phenotyping data in an automated manner. Excellent example of a solution to this problem was given in the work of Schmidt et al (Schmidt et al., 2023), where detecting and rectifying any potential phenotyping artifacts at an early stage was essential for conducting Genome-Wide Association Studies (GWAS) on a scale of nearly a thousand lines.

In the realm of plant phenotypic data preprocessing, a noteworthy tool is AllInOne Pre-processing (Yoosefzadeh Najafabadi et al., 2023), an open-source R-Shiny package that offers efficient solutions for data management. This package includes advanced features such as handling missing data, visualizing datasets, detecting outliers, estimating correlations, normalizing data, and conducting spatial analyses, all optimized for speed and user experience.

Building on this philosophy, our approach prioritizes extended longitudinal studies and integrates specialized methods for controlled environment time series analysis. StatFaRmer (Statistical Analysis for Farmers using R) is an open-source web tool that can be installed locally, requiring no prior knowledge of R.

Standard software bundled with phenotyping tools often falls short in providing user-centric interfaces for specific tasks, highlighting the need for a more intuitive design that meets diverse hardware and software requirements. The development goal for StatFaRmer was an enhanced data processing and insights generation in time-series data. The key requirements for the tool included:

1. Data Processing and Outlier Filtering: Implementing robust data processing techniques to filter out outliers and ensure the integrity and accuracy of the time-series data. The tool defaults to easily interpretable Z-score outlier detection, which can be switched to IQR outlier detection for less normal data by adjusting the options at the beginning of the main script. Outlier removal can be entirely bypassed by commenting out the “remove outlier groups” section of the main script. Additionally, the skewness and kurtosis of each selected group within the data can be tracked using the “Descriptive” tab in the StatFaRmer application.
2. ANOVA: Incorporating ANOVA analysis with *post-hoc* Tukey’s test to enable users to perform statistical comparisons and identify significant differences among various groups within the time-series data. The Shapiro-Wilk test and diagnostic plots are offered alongside ANOVA to evaluate normality and, thus, reliability of ANOVA results. The specific ANOVA model incorporates user-defined terms and is prominently displayed above the plot. While you can adjust the number of terms, only two-way interactions between them are considered to improve the clarity of the results.
3. Data Subsetting: Developing capabilities for data subsetting, allowing users to focus on specific subsets of the time-series data for more targeted and detailed analysis.
4. Factor Selection and Faceting: Enabling selection of phenotyping traits to facilitate in-depth analysis by grouping and examining data based on specific variables or factors.
5. Download Table with Statistics: Enabling users to download tables with comprehensive statistics, including selected grouping parameters such as gene, treatments, cultivar, and time clusters. This feature empowers users to access and utilize data insights offline for further analysis and reporting purposes.

Functionality and performance of the StatFaRmer tool was tested on plant data obtained using TraitFinder (Phenospex, Netherlands) (TraitFinder digital phenotyping workstation on wheels for lab- and greenhouse phenotyping automation - PHENOSPEX) high-performance phenotyping platform, complemented by our custom annotations of accessions.

## 2 Methods

### 2.1 Development of StatFaRmer

**Figure 1:**
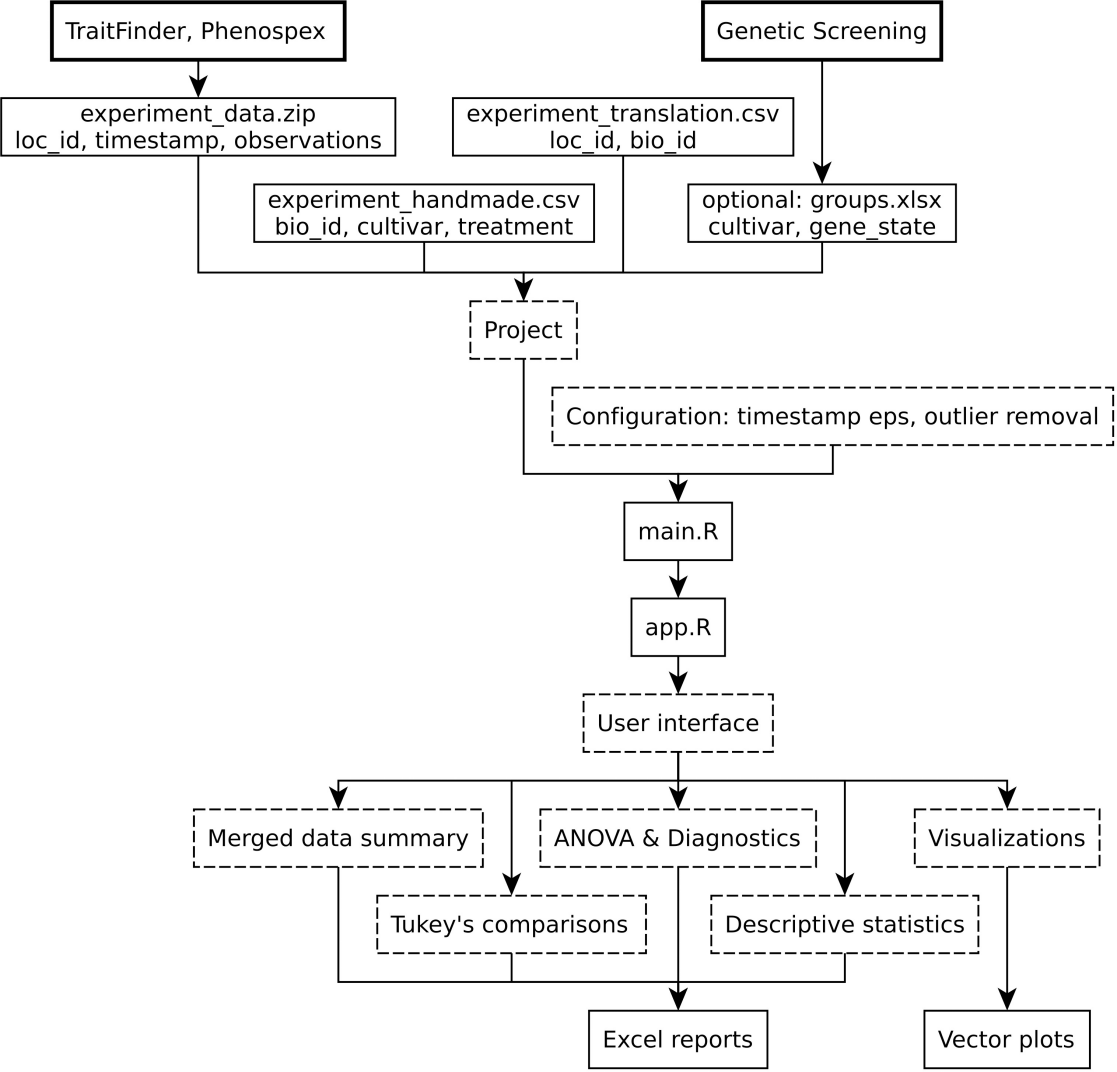
The overall block-scheme of the StatFaRmer platform. The block types are categorized: bold blocks represent required hardware and optional data sources, regular blocks indicate input and output files, and dashed blocks depict processing units and concepts.

StatFaRmer was developed fully with R (R Core Team, 2024) and consists of an initial processing script main.R, which can be modified to better work with a new experiment, and a shiny app.R, which allows users to visualize the data and quickly test a number of hypotheses, as shown in **Figure 1**.

The script main.R is equipped with functions that can handle various tasks using four groups of R packages:

- The first group consists of libraries that are primarily used for project stability and data validation: checkmate (Lang, 2017) for ensuring the validity of input data, logger (Daróczi, 2024) for testing tool’s interactivity, and renv (Ushey, 2023) for managing project dependencies.
- The second group includes tidyverse (Wickham et al., 2019) packages that focus on data manipulation and transformation: magrittr (Bache and Wickham, 2022) and rlang (Henry and Wickham, 2024) for simplifying code structure, purrr (Wickham and Henry, 2023) for functional programming, dplyr (Wickham et al., 2023a) for data manipulation, forcats (Wickham, 2023a) for handling categorical data, stringi (Gagolewski, 2022), stringr (Wickham, 2023b) and glue (Hester and Bryan, 2024) for string manipulation; readr (Wickham et al., 2024a), readxl (Wickham and Bryan, 2023) for reading tabular data, tibble (Müller and Wickham, 2023) for data structure, tidyr (Wickham et al., 2024b) for data tidying; with addition of janitor (Firke, 2023) for data cleaning, and lubridate (Grolemund and Wickham, 2011) for working with dates and times.
- The third group introduces statistical and clustering libraries: stats (R Core Team, 2024) for basic statistical functions and operations, and dbscan (Hahsler et al., 2019; Hahsler and Piekenbrock, 2024) for density-based clustering.
- astly, shiny (Chang et al., 2024) is a library for starting interactive web applications directly from R, providing a user-friendly interface for data visualization and analysis.

The script app.R utilizes various libraries to enhance its functionality: for dashboard creation, it employs shiny (Chang et al., 2024) with shinyWidgets (Perrier et al., 2024) for data wrangling, it incorporates tidyverse (Wickham et al., 2019) and vecsets (Witthoft, 2023); and for plotting, it features ggplot2 (Wickham, 2016). Additionally, libraries such as broom, flextable, moments, bslib, viridis, DT, multcompView (Komsta and Novomestky, 2022; Garnier et al., 2024; Gohel and Skintzos, 2024; Graves and Piepho, 2024; Robinson et al., 2024; Sievert et al., 2024; Xie et al., 2024) are used for general beautification and presentation of tabular results, while writexl (Ooms, 2024), svglite (Wickham et al., 2023b) are employed for data export. The script also includes publishing options through rsconnect (Atkins et al., 2024), facilitating tasks like data visualization, statistical testing, and data preparation.

### 2.2 Automated data handling

StatFaRmer imports user data from a specified project directory in the form of a TraitFinder-compatible experiment (.zip containing.csv files). The data includes plant coordinates (in the “unit” column) at each time point (in the “timestamp” column, ISO 8601 standard (ISO, 2017)), along with their respective numerical phenotypic parameters such as height and Normalized Difference Vegetation Index (NDVI).

Users are also required to provide two.csv tables: one named *_handmade.csv, which includes the required plant IDs (column “V.T.R,” indicating variety, treatment, and repetition number), as well as treatment and cultivar names (columns “Treatment” and “Cultivar”). This information will be displayed in the reports, overwriting any corresponding columns from the.zip file if provided. Additionally, a *_translation.csv table is necessary for establishing a one-to-one correspondence between plant IDs and unit coordinates, containing “V.T.R” and “T:X:Y” (should match TraitFinder ‘unit’ convention, indicating table number and spatial coordinates on it) columns.

An optional groups.xlsx table can be included, which must have a cultivar column and any additional columns that the user wishes to include as factors. Factor levels should consist of Roman letters, digits, and underscores for compatibility with the multcompView package.

On first access of StatFaRmer to the data, it determines time clusters. These clusters are introduced since the measurements of each plant take some time and timestamps for different plants are different for each experimental time point. For example, if the measurement takes 1 second, the timestamps for measurements at 2 PM for the first plant will be 2:00:00, for the second plant — 2:00:01, etc. Also, at this step the script identifies repeated measurements of each plant at one time point that have different timestamps as replicates, and separates them from measurements made at other time points. For this clustering, the script main.R of StatFaRmer uses DBSCAN clusterization algorithm with epsilon parameter. This parameter, defined in the main.R script in hours and currently configured to 1, efficiently processes experimental data irrespective of the plant measurement frequency. It is optimized to handle datasets where consecutive measurements are separated by an hour or longer. measurements with narrower time windows are considered technical repeats. From this point on, the original timestamps are replaced by “dbscan_cluster” times, which are close to the time points defined in experiment design, but do not coincide exactly. For example, the 2 PM time point in experimental design will result in timestamps of original data in the 2:00:00 — 2:05:30 interval, which become replaced by the median for all measurements in the cluster, say, 2:02:43 dbscan_cluster value.

Thus, “dbscan_cluster” times serve as a substitute for timestamp data, enabling faceting and factor selection in the analysis. It allows grouping timestamps with a given precision, which is required in an experiment with multiple consecutive scans. This step is necessary for further data processing and statistical analysis.

Then, all replicated measurements for plants (the measurements with the same dbscan_cluster time value) are filtered from outliers for each measured parameter (trait) within clusters based on a 3-sigma threshold. If the parameter is expressed as a percentage (for example, bins of specific ranges of NDVI values in TraitFinder datasets), then it is converted using the logit function, and placed in data tables as logit value for further analysis. Percentage values exactly equal to zero or one are replaced in advance with the nearest extreme finite values to avoid introducing infinities into analysis. This transformation was chosen as a more robust alternative to the arcsin, while still striving for interpretability (Lin and Xu, 2020). Additionally, the data table undergoes further modifications, such as column reordering, type conversions, the elimination of columns with only one factor level, and data reorganization for better readability. These manipulations are optional and can be controlled by the user as needed. However, this functionality is currently implemented by commenting out the relevant sections of the main script. At this stage, additional criteria for grouping are incorporated based on the groups.xlsx file located in the current project directory. Users also have the flexibility to group plants based on additional criteria in accordance with the experimental design (e.g., control, factor treatment), as demonstrated in section 2.4. All of these steps are implemented to prevent collisions during the subsequent ANOVA. Then StatFaRmer computes medians for technical repetitions of the same plants within time clusters, and saves the processed table as an RDS file for the Shiny app.

StatFaRmer performs ANOVA on user-selected grouping factors and their interactions for a specified trait, followed by Tukey’s test. It includes Shapiro-Wilk tests to assess normality, displaying results and diagnostics in the ANOVA tab to aid informed decisions on further analysis, such as adjusting outlier removal strategies and model parameters.

### 2.3 User interface

StatFaRmer has a friendly interface made with the Shiny app framework, which allows users to analyze data interactively and share the dashboards. Users can choose grouping factors, factor levels, treatments, and more to customize their analysis. The main panel displays faceted violin plots with automatically assigned characters from multiple comparisons, based on ANOVA/Tukey’s tests of user defined groups. There are also tabs for looking at raw data, stats, ANOVA and Tukey’s test results, and group comparisons, giving users more ways to analyze their data.

The tool’s server logic handles data smoothly using reactive expressions and debouncing techniques. It allows users to select and change variables by specifying them in a drop-down list and removing them using the Backspace or Delete keys for dynamic data visualization. By presenting mean, median, deviation from normal distribution, ANOVA and Tukey’s test results, StatFaRmer provides a comprehensive view on the data. Additionally, StatFaRmer makes it easy for users to see and download plots as SVG files, formatted tables with formulas used, raw data and their stats, ANOVA results, Tukey’s test outcomes, and group comparison characters. This feature makes it simple for users to explore, interpret and record the results of their statistical analysis.

### 2.4 Utilization of the StatFaRmer tool for statistical analysis of phenotypic data

The tool is run in a browser (tested on major web browsers) at address (https://stathmin.shinyapps.io/StatFaRmer). A sample dataset of different plant species (bread wheat (*Triticum aestivum*), durum wheat (*Triticum durum*), and triticale (× *Triticosecale*)), cultivars (35 variants) and plant genotypes (allelic state of 3 genes), with different treatments (3 variants), and the time series of morphological and spectral parameters of these plants is loaded in this tool as an example and available on GitHub.

## 3 Results

### 3.1 Initial data processing

#### 3.1.1 The application of the DBSCAN method for the soybean dataset

Clustering similar timestamps with DBSCAN — with epsilon guided by the experimental design (e.g., event duration or data collection frequency, typically 1 hour) allows for identification of patterns in time-series data. This approach minimizes artifacts from cruder timestamp aggregation, clarifying meaningful relationships. The effectiveness of DBSCAN is contingent on selected epsilon, requiring meticulous tuning for optimal clustering.

**Figure 2:**
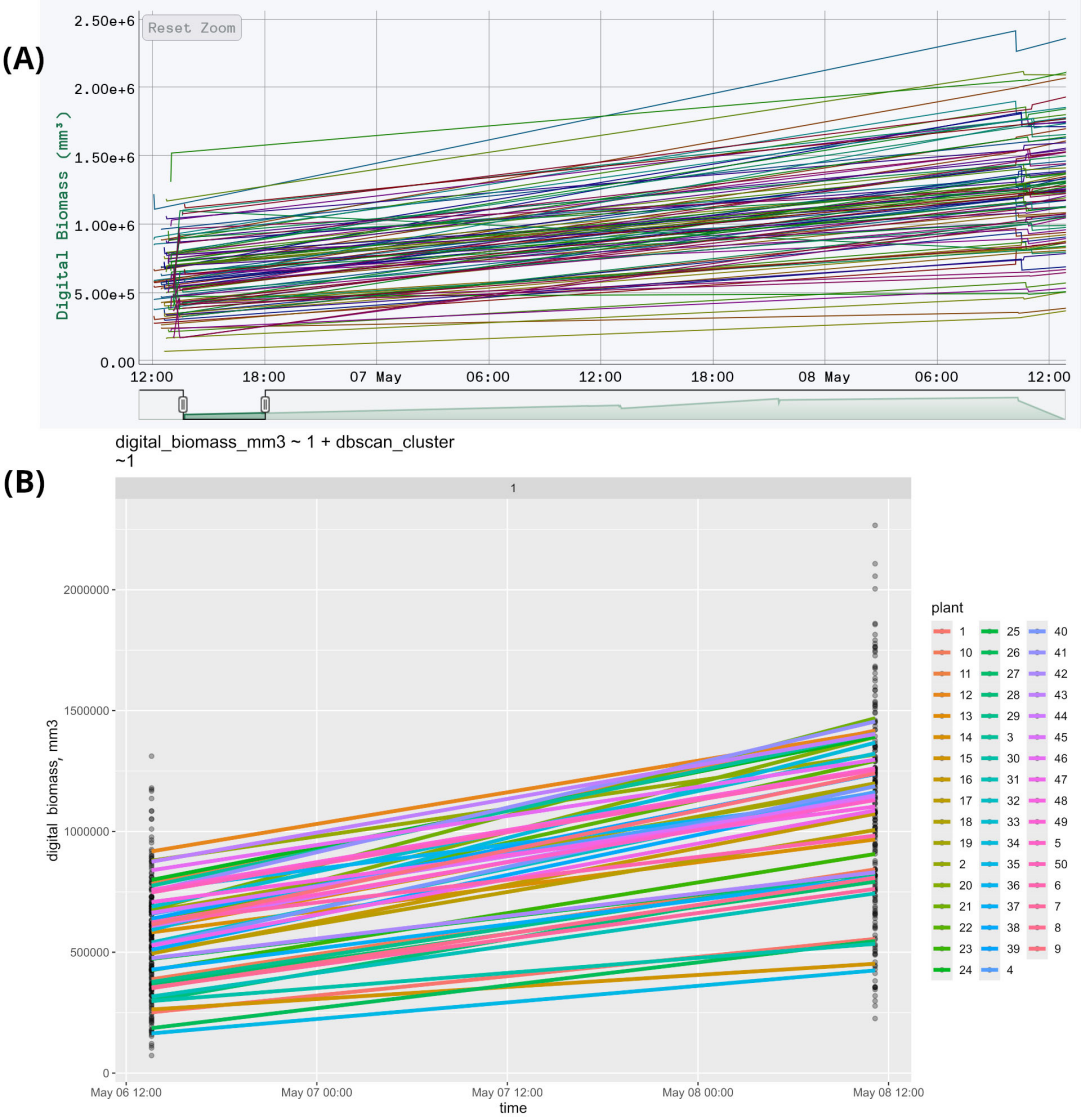
**(A)** The first two measurements of soybean phenotyping. The X-axis represents the date and time of each scan, while the Y-axis shows the corresponding digital biomass. Each line on the graph corresponds to an individual plant, resulting in a total of 200 graphs. The data is unprocessed, with each timestamp represented as a separate plotted point, totaling 34 points per measurement. This creates visual clutter, evident as “ladders” at the edges, and complicates further analysis. **(B)** The same data, presented in StatFaRmer. The repetitions are averaged and displayed as one graph per variety, reducing the total to 50 graphs in one image. Similar timestamps are clustered and represented as single time points, enhancing the graph’s visual accessibility and making the data easier to use for statistical analysis. The points in this graph represent unprocessed measurements, while the lines represent data processed in StatFaRmer.

This feature is exemplified by an experiment on soybean (*Glycine max*) phenotyping, where 50 varieties were grown under two photoperiods with two repetitions, totaling 200 plants. Seed preparation involved treating dry seeds with a fungicide, then treated soybean seeds were planted 2 cm deep in 500 ml pots filled with 230 g of moistened peat, with four seeds per pot. After germination, three plants were retained per pot. Plants were grown in a climate chamber under a photoperiod of 22 hours light and 2 hours dark. Initial lighting was continuous (24/0) for the first three days to prevent seedling stretch. Temperatures were maintained at +26°C daytime and +25°C nighttime, with an intensity of 400 μmol/m²/s. Pots were arranged randomly and repositioned weekly. During the first 7-10 days, plants were watered with room-temperature water, then with 50 ml as needed. Once true leaves appeared, a mineral fertilizer was applied daily at 30 ml per pot. For scanning, plants were moved to a phenotyping table, organized by variety and replication. Soybeans were grouped into sets of 12 pots, recorded in separate blocks, totaling nine blocks. **Figure 2A** displays the raw data in HortControl (PHENOSPEX, [[NoYear]]), the default application of Phenospex, while **Figure 2B** presents the same data after time clustering using StatFaRmer.

Biologically, this function is crucial for making the data more accessible to humans. In the original unprocessed figure, the volume of data is too large to discern any trends (200 graphs compared to 50). Additionally, the spikes and “ladders” caused by the absence of timestamp clustering make it difficult to follow the individual graphs and the figure as a whole.

#### 3.1.2 Example of the outlier removal for bread wheat, durum wheat, and triticale datasets

Filtering outliers in timestamp clusters using a 3-sigma threshold preserves data integrity in time-series analyses. This method discards measurements beyond three standard deviations from the mean, mitigating measurement errors and highlighting true trends. It assumes normality; thus, alternative methods like interquartile range (IQR) may be needed. In this case, the variable use_IQR changes the filtration method.

**Figure 3:**
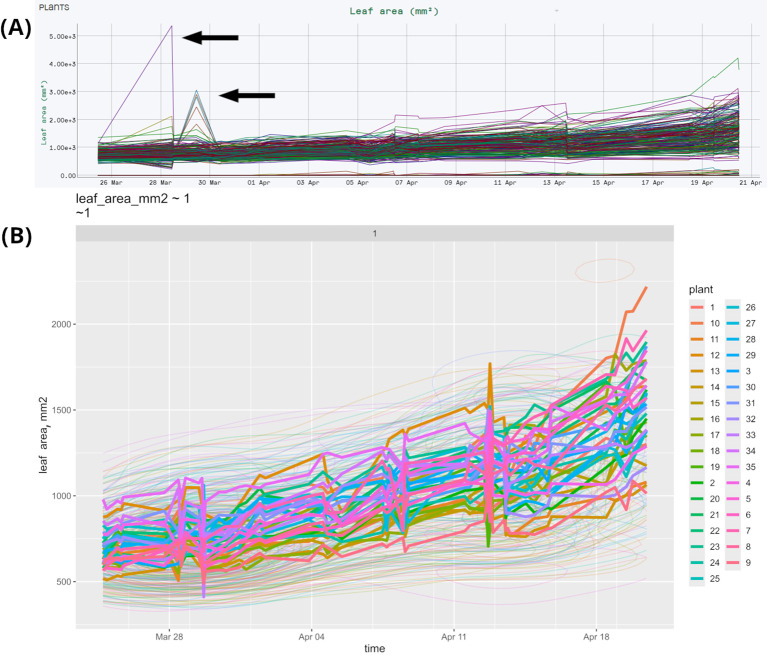
Plots of the same data collected during bread wheat, durum wheat, and triticale phenotyping from HortControl and StatFaRmer. **(A)** Unprocessed data collected during phenotyping by HortControl, the primary software for the TraitFinder platform. The X axis is the date and time for each scan, the Y axis is the corresponding plant leaf area. Each line on the graph represents an individual plant. **(B)** Comparative visualization from StatFaRmer with individual lines displaying the changes over time of DBSCAN cluster medians. The outliers are removed, the spike at Apr-13 is made more evident, presuming technical issues to be studied in more detail. The thin lines represent the “height map” or 2D density map and are used when the number of plotted observations exceeds 2000 individual points.

This method is illustrated by our sample dataset of bread wheat, durum wheat, and triticale plants featuring 35 varieties and 3 treatments, measured across 2 repetitions, totaling 210 plants. A more detailed description of this experiment can be found in section 3.5. The original dataset includes outliers, as shown in **Figure 3A**, where specific outlier measurements are indicated by arrows. StatFaRmer automatically filters these outliers, resulting in the adjusted dataset shown in **Figure 3B**. After applying either 3-sigma threshold or IQR outlier removal, 99% and 97% of measurements are retained, respectively. The outliers are primarily caused by uncontrollable external factors, such as improper positioning of glossy or reflective plant parts or interference from nearby foliage in the scanning area. The tool allows analyzing the affected plants in subsequent timestamps to assess any persistent issues or trends.

The biological significance of this lies in the automatic removal of obviously unrealistic measurements that result from errors, such as interference from a person or an inanimate object during measurements. Since we conduct numerous large-scale experiments in limited spaces, we have encountered instances of human error, such as rearranging pots while TraitFinder is still running. While we document these incidents thoroughly, having a function that removes the most extreme outliers is essential.

#### 3.1.3 Example of logit transformation for sugar beet dataset

Logit transformation corrects deviation from normality in percentage data, particularly near 0% and 100%, by converting bounded proportions to an unbounded scale, stabilizing variance for statistical analyses like linear regression and ANOVA while still being easily interpretable (Lin and Xu, 2020). Before the transformation, we replace 0% or 100% values with the closest observed values to maintain the integrity of the analysis.

As an example of such a percentage data, which normally occurs in phenotyping analysis, we have studied the share of specific Plant Senescence Reflectance Index (PSRI) bins. PSRI is calculated as follows:


$$\frac{RED-GREEN}{NIR}$$

where the red wavelength is 620-645 nm, green is 530-540 nm, and near-infrared (NIR) is 720-750 nm. Spectral indices can also be represented using bins. A bin counts how many points of a given 3D scan fall within its defined boundaries, each having a lower and upper limit. The number of points within the bin is expressed as a percentage of the total area. In this case, the PSRI index consists of six bins, with bin 0 [-4:-0.8] being the lowest. A lower PSRI value indicates healthier plants (Merzlyak et al., 1999).

To illustrate this feature we picked an experiment with sugar beet (*Beta vulgaris*) plants of 2 varieties which were exposed to a prolonged period of cold (vernalization) at 5 and 10°C (24 plants in total) and were grown at lighting conditions differing by spectrum. After pre-sowing preparation, the seeds for germination were placed on moist filter paper in a plastic container, which was covered with film and kept in the dark at room temperature for three days. The growing containers used were 500 ml plastic seedling pots filled with sterilized peat that had been treated for 15 minutes at 121°C, mixed with perlite in a 5:1 ratio. Germinated seeds were planted at a rate of three seeds per pot. Vernalization was carried out at temperatures of +5°C and +10°C. Temperature was monitored through temperature and humidity sensors the entire duration of the experiment. Plants were grown under LED lamps with photoperiods of 22/2 and 10/14 hours, using white light at an intensity of 60, blue + red light at an intensity of 500, and blue + red light at an intensity of 466.

**Figure 4:**
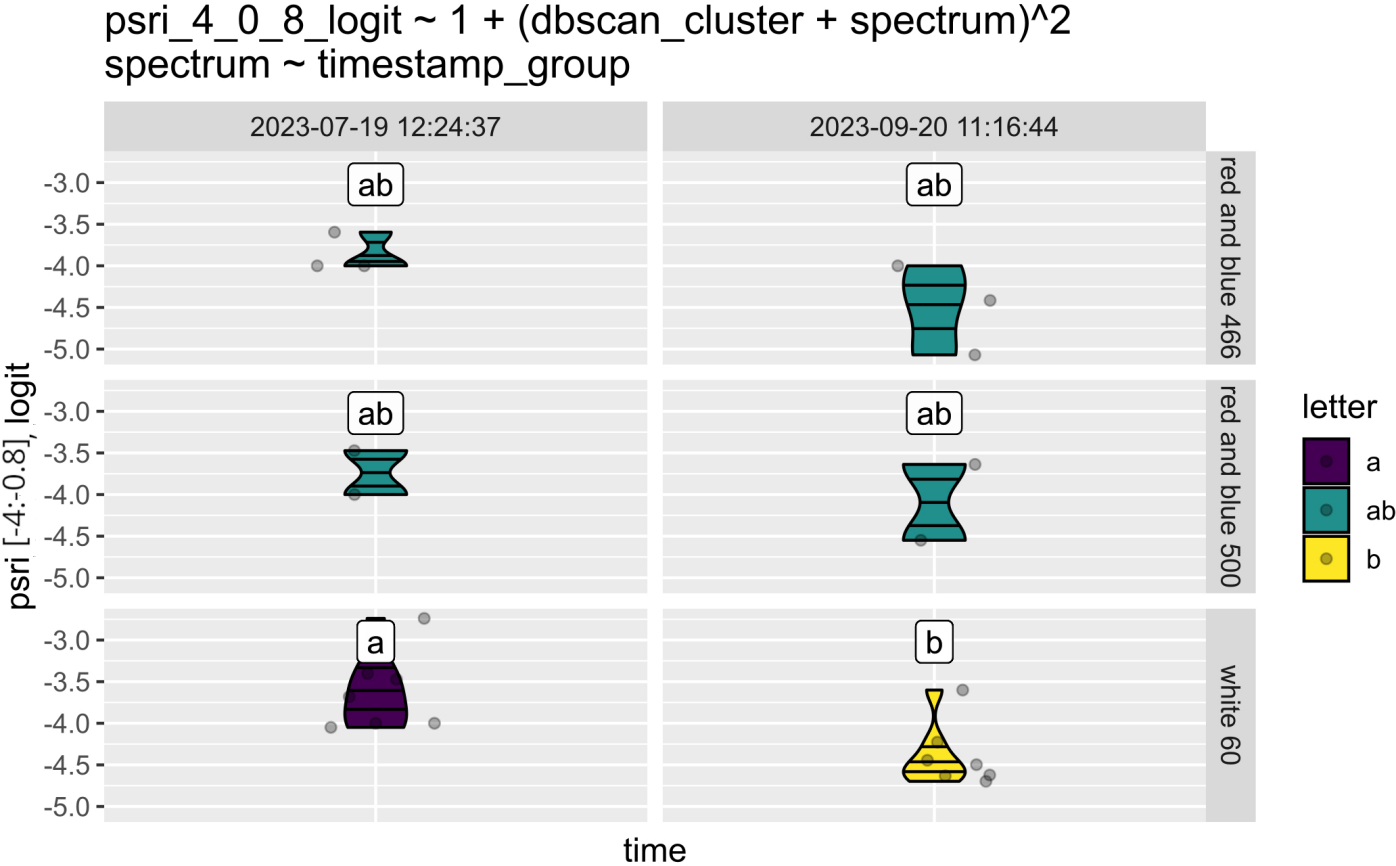
Results of the Plant Senescence Reflectance Index (PSRI) for sugar beet plants after a vernalization period, under different lighting conditions in the [-4:-0.8] bin. This bin is the lowest, with lower PSRI values corresponding to healthier plants. The graph comprises six panels for easy comparison among the different conditions. The left three panels display measurements from the initial time point, while the right three were measured two months later to evaluate how the spectrum affects the plants. On the right, there is a bar indicating the spectrum options. The colors represent different clusters. Clusters that do not share common characters (a and b) are significantly different. The equation above the panels represents the analyzed factors and interaction in the ANOVA.

In **Figure 4** we compare the PSRI [-4:-0.8] bin of plants grown at various lighting conditions at temperature 10°C. The Plant Senescence Reflectance Index (PSRI) is a key parameter in plant science for assessing leaf senescence. Proposed in 2002 (Merzlyak et al., 1999), it helps estimate the onset, stage, relative rates, and kinetics of senescence and ripening processes.

As shown in **Figure 4**, the lowest and healthiest bin of PSRI was significantly reduced after two months of growing the plants under white light, while there was little change under the other conditions. Based on the conducted research, it can be stated that a short day, particularly in combination with low temperature, significantly slows down plant development. However, the blue-red spectrum at an intensity of 400-500 μmol/m²/s mitigates this effect.

#### 3.1.4 Example of support for supplementary data for bread wheat, durum wheat, and triticale dataset

Integrating Phenospex (TraitFinder) data with user-generated tables enriches datasets by adding variables like environmental and genetic factors, enhancing analysis robustness and validity, and improving insights into phenotypic traits and research reproducibility. Grouping and subsetting data by genes, treatments, cultivars, and time clusters enables targeted hypothesis testing for selected traits.

**Figure 5:**
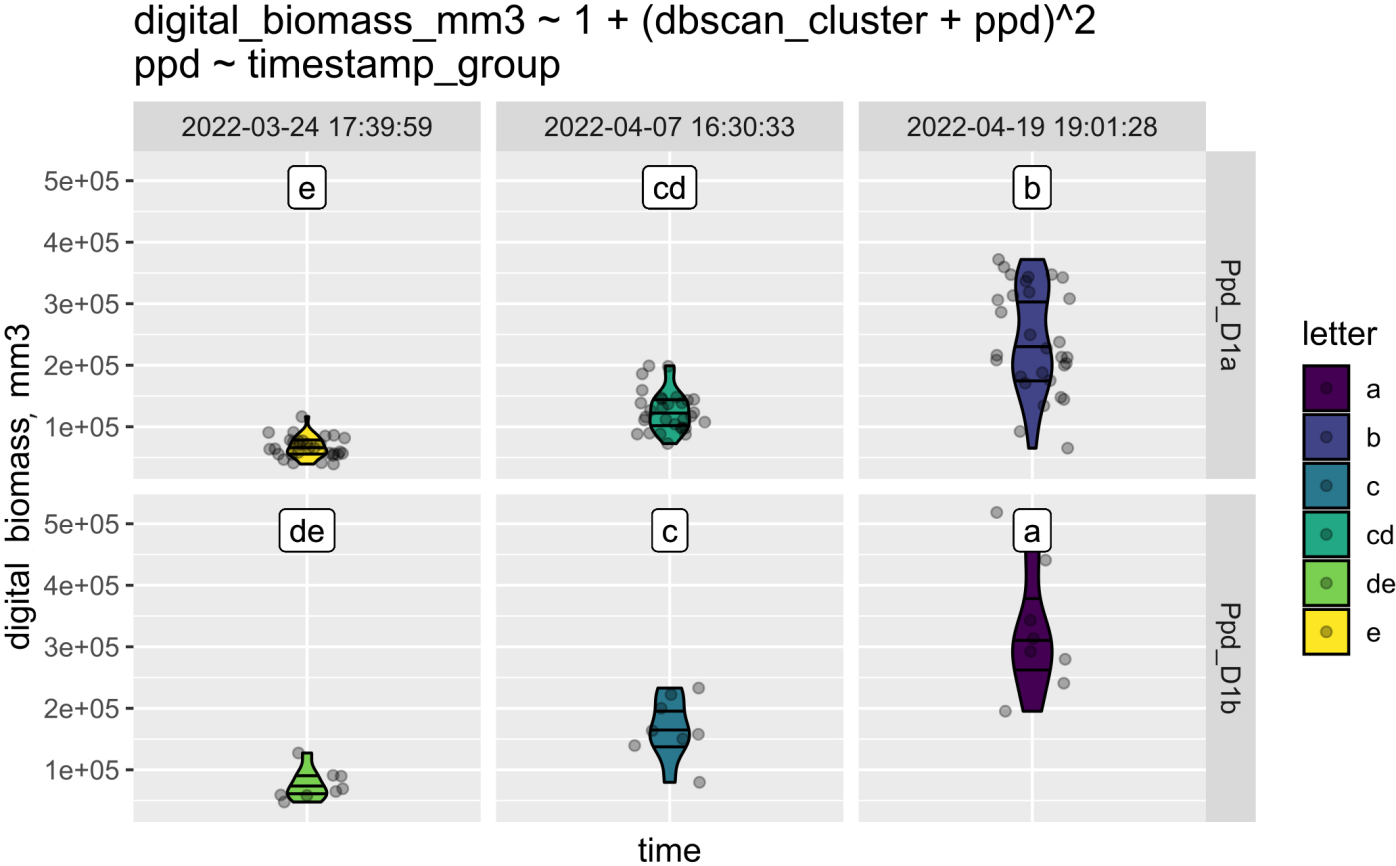
This graph consists of six panels: the top three illustrate how the digital biomass of plants with the *Ppd-D1a* allele changed over time, while the bottom three show the same process for plants with the *Ppd-D1b* allele. The three time points are indicated at the top, and colors represent the respective clusters. Clusters sharing a common character (c and cd; cd and de; de and d) may overlap. They are arranged in order of digital biomass value, with plants in cluster “a” having the highest biomass and being the most distinct from those in cluster “e.” The equation above the panels represents the correlation of the analyzed factors in the ANOVA test.

This step is illustrated with a subset of our experiment with bread wheat. All the cultivars of bread wheat were screened for their allelic states of the *Ppd-1* gene, which is known to regulate inflorescence architecture and paired spikelet development in bread wheat (Boden et al., 2015). A more detailed description of this experiment can be found in section 3.5. After the experiment was completed, the data were uploaded in csv format and supplemented with information about *Ppd-1* alleles in each cultivar. Using StatFaRmer, we assessed the effect of the *Ppd-1* alleles *Ppd-D1a* and *Ppd-D1b* on the digital biomass of bread wheat (**Figure 5**). Photoperiod-insensitive alleles of *Ppd-1* are commonly utilized in breeding to reduce the requirement for long day lengths and promote earlier flowering in the season. The graph indicates that the digital biomass was nearly equal for plants with *Ppd-D1a* and *Ppd-D1b* alleles at the beginning of the experiment (first column). However, by the end of the experiment (third column), plants with the *Ppd-D1b* allele exhibited significantly higher digital biomass, suggesting that some *Ppd-1* alleles may enhance biomass gain.

### 3.2 Examples of phenotypic data visualization with dynamic faceting for sugar beet, corn and sunflower datasets

The tool’s visualization allows for interpretation of temporal variations among groups, marked by characters, obtained based on Tukey’s test p-values, where common characters identify levels or groups that are not significantly different. This aids in hypothesis formulation and decision-making. Alternatively, it allows users to observe and compare time trends between groups of interest. A color-coded, color-blind-friendly palette enhances accessibility and clarity for all users.

**Figure 6:**
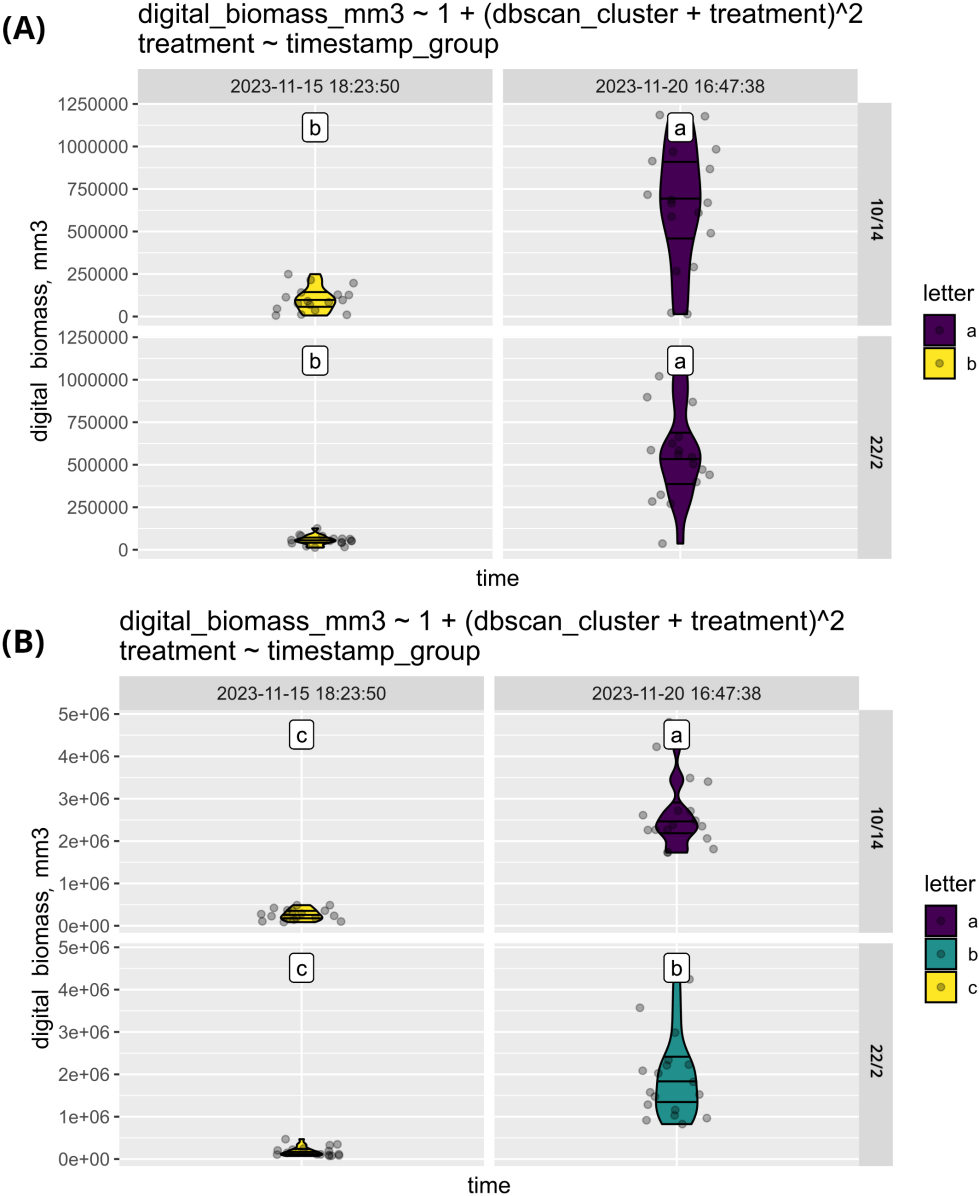
The comparison of biomass growth of sunflower **(A)** and corn **(B)** under two distinct sets of conditions. The graph illustrates that while corn exhibited variability in response to the different growing conditions, sunflowers showed consistent growth across both environments.

This function is illustrated (**Figure 6**) with a dataset consisting of plants of 3 varieties of corn (*Zea mays*) and 3 varieties of sunflower (*Helianthus annuus*) in 6 repetitions grown under two different conditions (72 plants in total). The sunflower varieties used were Zhemchuzhina, Korona, and CC-4, while the corn varieties included Marmeladka, 147MB, and 975-5. The plants were grown under photoperiods of 10/14 and 22/2.

As shown in **Figure 6**, digital biomass is similar across various conditions for both sunflower and corn at the initial measurement. At the second time point, while sunflowers continue to exhibit similar digital biomass across all treatments, corn demonstrates a preference for the 10/14 photoperiod. This suggests that photoperiod has a more significant impact on corn compared to sunflower. Alternatively, the effects of photoperiod on sunflowers may manifest later due to differences in growth rates.

The facet syntax aligns with R formula principles, allowing grouping variables to be placed around the tilde (~) for distinct vertical or horizontal subplots. This flexibility enhances data clarity and interpretability, while using “~.” simplifies visualizations by removing faceting when necessary.

**Figure 7:**
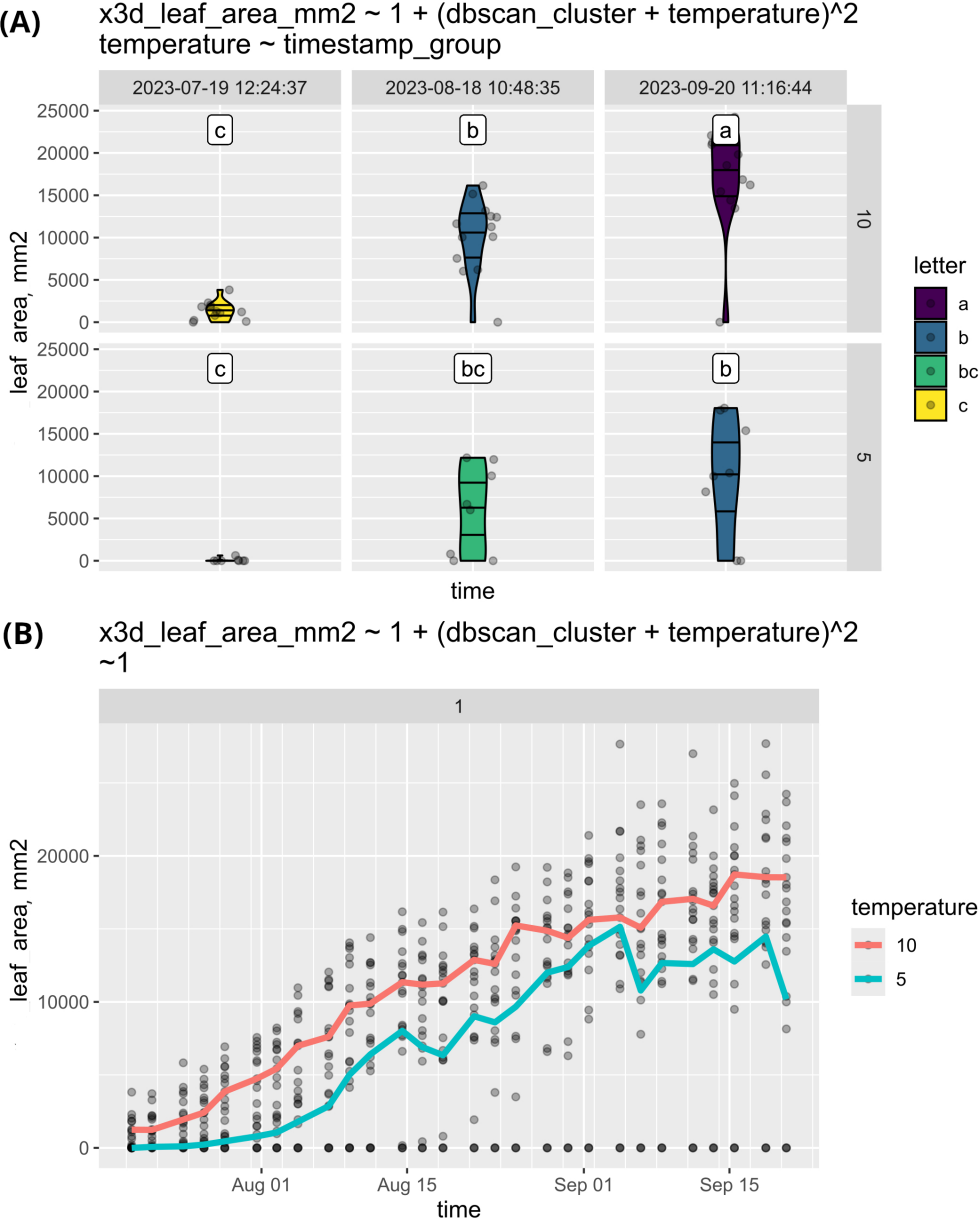
**(A)** This study compares the leaf area growth of sugar beets vernalized at 5°C and 10°C. The results indicate that plants vernalized at 10°C exhibited a larger leaf area compared to those vernalized at 5°C. However, the observed distributions are not normal, raising questions about the underlying factors affecting growth. Additionally, the same data was visualized as a timeline **(B)** by eliminating the faceting. The deviations from normality observed in **(B)** can thus be attributed to some plants not initiating growth throughout the experiment. The data is plotted in a single subplot by setting the faceting formula to “~ 1” and using genotype as the grouping factor.

To illustrate this feature we used the previously mentioned experiment with sugar beet plants and compared the growth of leaf area with different temperatures of vernalization. As shown in **Figure 7**, the leaf area of plants exposed to 5°C vernalization remained consistently lower throughout the experiment. This likely indicates that the plants experienced more stress, resulting in suppressed leaf growth. Conversely, it may suggest that the plants were conserving energy to invest in flowering.

### 3.3 Statistical evaluation of the differences between groups for significance

#### 3.3.1 Example of descriptive statistics for cocklebur and lettuce datasets

StatFaRmer reports raw tables and tables with descriptive statistics. They provide essential insights on sample size (n), central tendency (median, mean), variability (cv_perc), range (min, max), and distribution shape (skewness, kurtosis), guiding further analysis.

This feature is illustrated with an experiment in which 22 plants of cocklebur (*Xanthium strumarium*) and 22 plants of lettuce (*Lactuca sativa*) were treated with 4 different herbicides. Plants were grown in pots measuring 16.5 cm x 9.5 cm x 8.5 cm, with two plants per pot, using a universal soil that contains all the necessary macro- and microelements.Soil moisture was maintained at 50% through watering three times a week, and plants were kept indoors at 22°C and 60% humidity with 16 hours of light. Temperature and humidity were regulated using air conditioning units and water containers, and they were continuously monitored with temperature and humidity sensors throughout the entire duration of the experiment.

On day 0, plants were sprayed with water or clopyralid formulations (20.6 mL/m²) in five replicated pots. Clopyralid formulations, Hacker WG and Hacker 300 SL, were obtained from JSC August Inc. A gemini surfactant, 16-6-16, was synthesized from hexadecyl bromide and N,N′-tetramethylhexamethylenediamine. Readers can find a more detailed description of the results in our article dedicated to this experiment (Mirgorodskaya et al., 2023).

**Figure 8:**
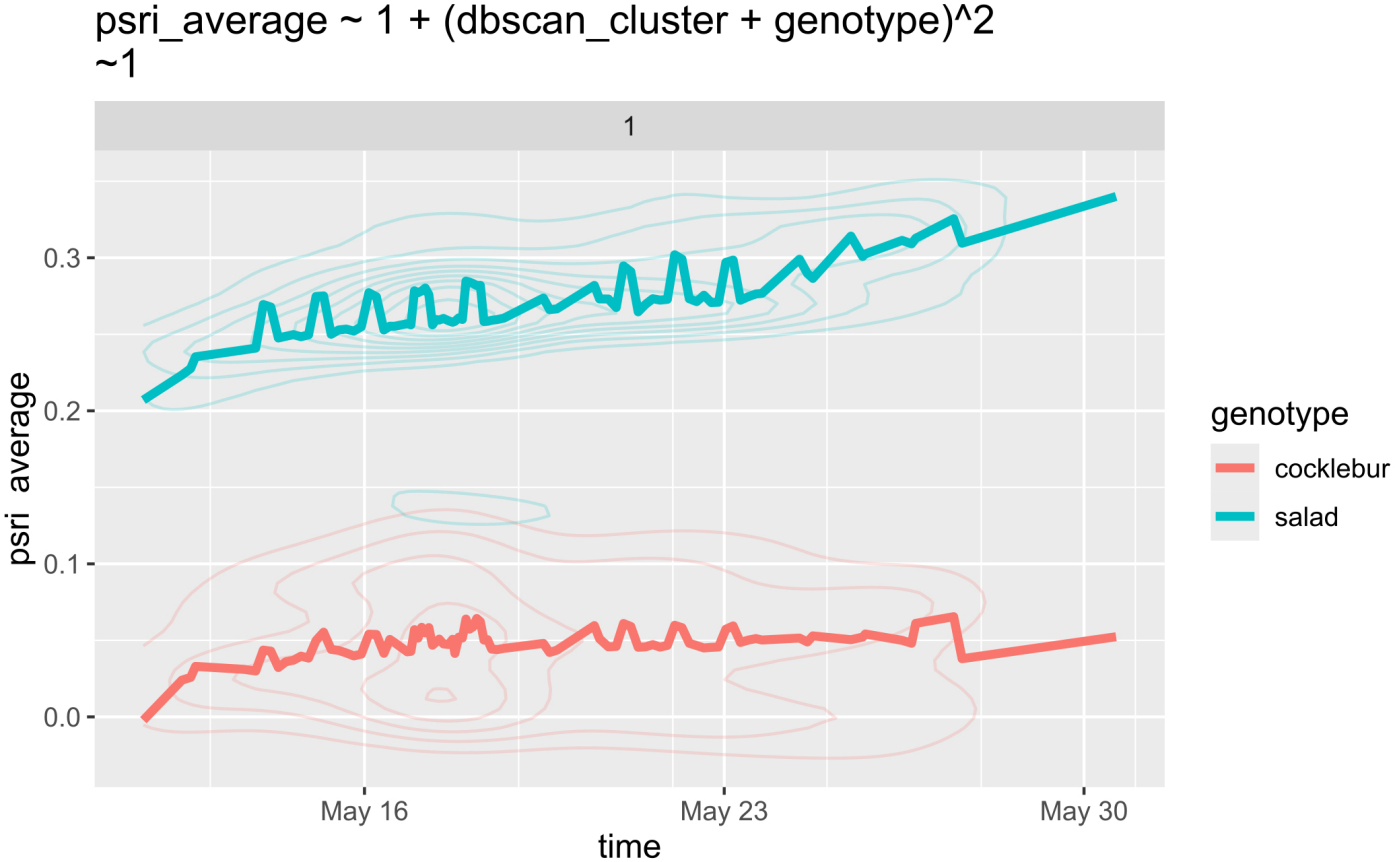
Timeline representation of the herbicide experiment with lettuce and cocklebur. Due to over 2,000 observations, the data is presented as density maps. Notably, May 17 exhibits a decline in average PSRI for one cocklebur sample, lasting for several days.

**Figure 8** demonstrates that lettuce plants were significantly more susceptible to herbicide treatment compared to cocklebur, as indicated by the higher PSRI in lettuce. The descriptive statistics in **Table 1** confirm this observation.

**Table 1:** Descriptive statistics from the herbicide experiment at three time points.

	group	n	median	mean	cv_perc	min	max	skewness	kurtosis
1	1:cocklebur	20	0	0.01	-1291.42	-0.03	0.05	0.32	1.62
2	69:cocklebur	10	0.06	0.07	75.2	0.01	0.15	0.21	1.72
3	35:cocklebur	20	0.05	0.13	383.78	-0.07	0.55	1.07	2.79
4	1:lettuce	20	0.21	0.21	13.64	0.15	0.25	-0.17	2.07
5	69:lettuce	10	0.28	0.28	14.35	0.18	0.33	-1.48	5.18
6	35:lettuce	20	0.34	0.3	39.23	0	0.41	-1.74	4.42

The “group” column represents the combination of grouping factors—plant type and time point— where 1 indicates the start, 35 the second time point, and 69 the experiment’s conclusion.

The increased sensitivity of lettuce indicates a deficiency in protective mechanisms against herbicides, making it more susceptible to chemical stress. In this experiment, lettuce was used as a control plant due to its low resistance to chemical stress, and its higher PSRI indicates that the herbicide is effective.

#### 3.3.2 Example of ANOVA and Tukey’s test for cocklebur

ANOVA’s user-selected factors are automatically supplemented with their two-way interactions, which allows for thorough assessment of variable influences on responses. This aids in identifying complex relationships, but researchers must be cautious of potential overfitting due to increased model complexity. StatFaRmer reports post-ANOVA tables (**Table 2**) to succinctly present key results.

**Table 2:** ANOVA table illustrating the significance of observed effects in cocklebur plants treated with four herbicide compositions across days 1, 2, and 10 of the experiment.

	term	df	sumsq	meansq	statistic	p.value	sig
1	dbscan_cluster	2	0.16	0.08	96.75	0	***
2	treatment	3	0.02	0.01	8.89	0	***
3	dbscan_cluster:treatment	6	0.01	0	2.13	0.07	.
4	Residuals	48	0.04	0			

It includes statistics such as degrees of freedom (df), sum of squares (sumsq), mean squares (meansq), test statistic, p-values, and significance levels, where *** signifies ‘p-value< 0.001’ and. indicates ‘p-value > 0.05’.

**Figure 9:**
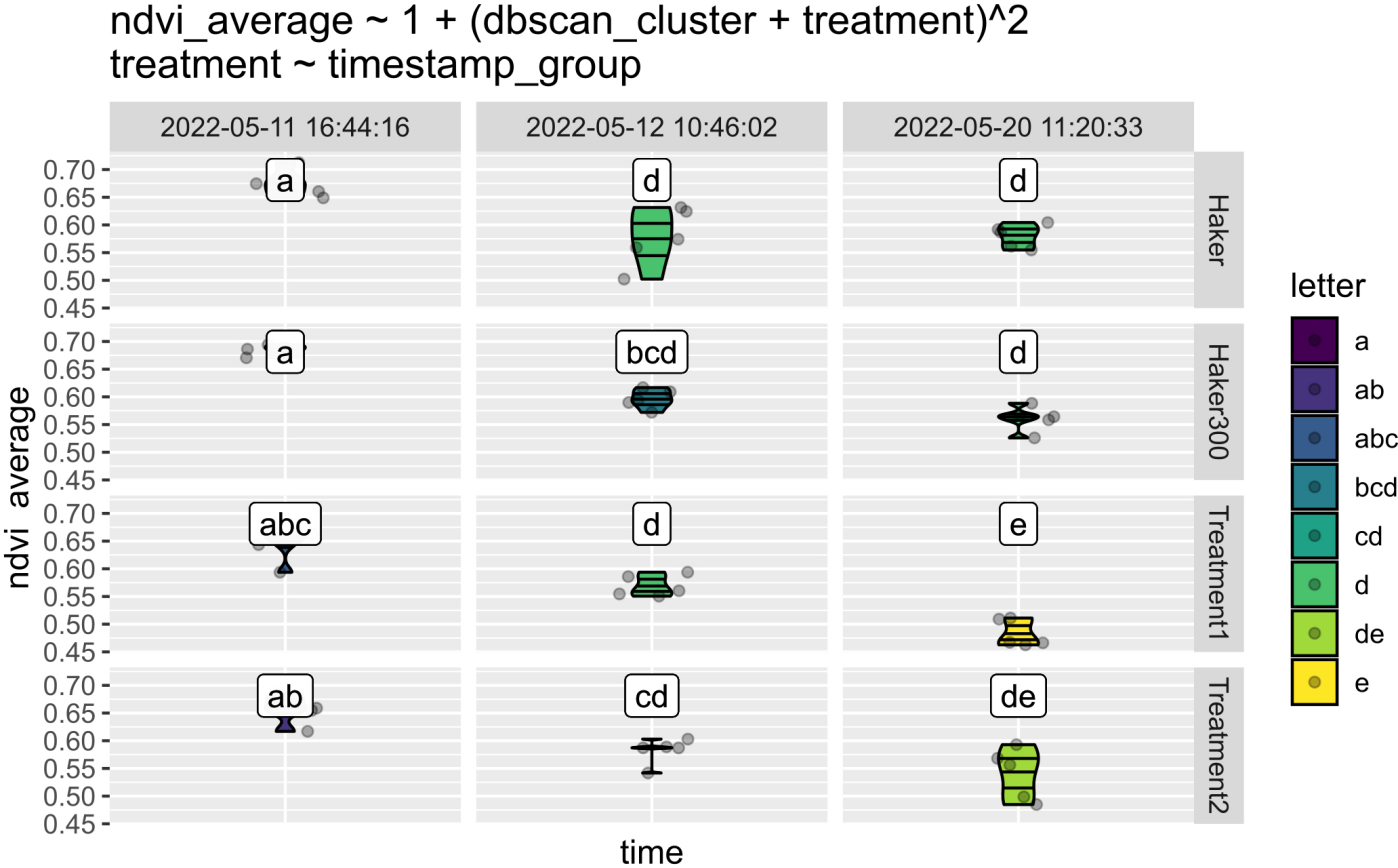
Comparison of NDVI in cocklebur plants treated with various herbicides. Treatment 1 notably decreased NDVI, showing significant effects the day after application and lasting for a week. The colors represent clusters, where lack of common characters in cluster names indicate statistically significant differences between the clusters. These lowercase letters are automatically assigned characters from multiple comparisons, based on ANOVA/Tukey’s tests of user defined groups. They are used consistently across multiple figures.

This feature is illustrated by a subset of the same experiment. The Normalized Difference Vegetation Index (NDVI) is a widely used vegetation index for assessing plant health. **Figure 9** displays sets of three time points for plants treated with four different herbicide compositions. Cluster “a” represents higher NDVI values, indicating healthier plants before herbicide treatment. Conversely, cluster “e,” which has the lowest NDVI, appears only at the end of the experiment with Treatment 1. This suggests that this treatment is particularly effective at destroying this specific weed.

The “Tukey” feature identifies contrasts among parameter combinations across factors and time points. StatFaRmer reports these tables after the Tukey’s test to present key results, including contrasts, estimates, confidence intervals (conf.low, conf.high), adjusted p-values, and significance (sig).

### 3.4 Results export

In StatFaRmer Shiny App, all tables and produced plots can be downloaded after applying filters and subsets (via the “Download Full Results” and “Save Plot as SVG” buttons).

### 3.5 The dataset of high-throughput plant phenotyping of *Triticeae*

**Figure 10:**
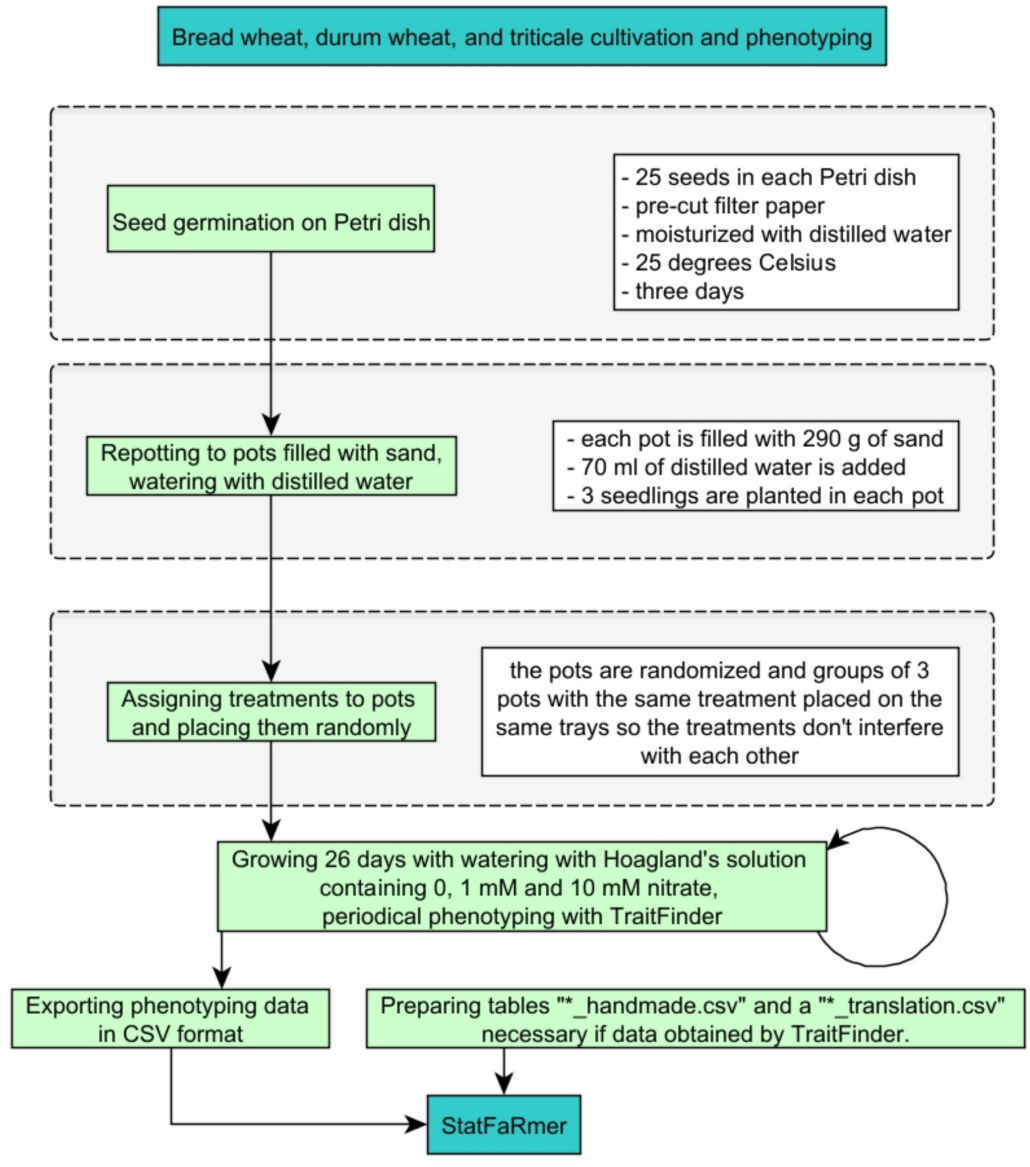
Schematic representation of the study examining nitrate’s effect on bread wheat, durum wheat, and triticale growth, which produced the sample dataset for StatFaRmer. * here represents "wildcard character".

To evaluate the features and performance of StatFaRmer, a study on the growth of various cereal plants under conditions of nitrogen starvation and low and high nitrate concentrations has been performed (**Figure 10**). The grains of different cultivars of bread wheat (*Triticum aestivum*), durum wheat (*Triticum durum*), and triticale (× *Triticosecale*) were placed on Petri dishes containing moist filter paper and incubating them at 25 degrees Celsius. After seed germination, the seedlings were transferred to pots filled with sand and watered regularly with modified Hoagland solutions with various concentrations of nitrates. These solutions contained 0, 1mM and 10mM potassium nitrate, and the first two solutions were supplemented by potassium chloride to maintain the same molar potassium content that the third solution. The pots were arranged randomly. The modified Hoagland solutions were added 3 times per week in quantities to replace the lost weight of the pots. Temperature and humidity were monitored throughout the entire duration of the experiment.

Phenotypic observations of all plants were conducted three times daily in two to three replicated measurements using the TraitFinder phenotyping platform (Phenospex, Netherlands). This platform is based on a PlantEye — a laser scanner coupled with a multispectral imager. PlantEye generates a 3D point cloud with reflectance values for each point at four wavelengths. The TraitFinder we used was equipped with two PlantEye scanners that were installed at some distance and angles to minimize plant blocked areas. Two 3D point clouds from each PlantEye were combined into one point cloud with better coverage of plants. Using these 3D point clouds, a HortControl software calculated various morphological and spectral parameters of the plant.

Among the morphological parameters are plant height, leaf area and digital biomass, which is determined by the product of the two previous parameters and for plants with the same architecture correlates well with the real plant biomass (Vadez et al., 2015; Maphosa et al., 2017; Quijia Pillajo et al., 2024). Among the spectral parameters are NDVI and its “bins” — the proportion of plant leaf area that has NDVI values in a certain range. A set of parameters obtained during the experiment for all plants and time points exported in archived CSV format, was combined with annotation tables and imported into StatFaRmer as a standard sample dataset.

### 3.6 StatFaRmer performance evaluation

Data processing speed of StatFaRmer has been tested on a laptop equipped with an AMD Ryzen 5 5500U with Radeon Graphics 2.10 GHz with 8 GB of RAM. The test showed that the initial launch of the program using the bread wheat, durum wheat, and triticale study dataset (18 Mb.csv file that contains 58,380 rows and 49 columns) takes 30 seconds. Subsequent launches take only 4 seconds, since the.rds objects used by the shiny app were already created when the application was first launched.

ANOVA analysis and plotting violin charts for a phenotypic parameter for selected time points occurs almost instantly for numbers of treatment and timestamp levels below ten. Plotting time series trends for big datasets such as the bread wheat, durum wheat, and triticale study dataset (almost 20,000 measurements to represent after averaging within DBSCAN clusters) was sped up by drawing density maps instead of point geometric objects when trying to plot more than 2,000 measurements.

In this series of studies, StatFaRmer has become essential to evaluate the outcomes obtained from digital time-series phenotyping due to its flexibility and a wide range of customizable parameters for analysis. During our work with this tool, we were able to explore a diverse range of plant cultivars and identify the factors that influence the condition of specific plants. For instance, in our latest experiment, we grew plants of various varieties, and because of this tool, it was possible to not only compare the growth patterns between different varieties but also assess the impact of various alleles by grouping the varieties based on these allelic variants.

The resulting tool can be accessed at 9https://github.com/Stathmin/StatFaRmer), with the instructions on installation and the sample dataset provided.

## 4 Discussion

The evaluation of StatFaRmer underscores its efficiency in processing data for digital time-series phenotyping, with an initial launch time of 30 seconds, followed by just 4 seconds for subsequent access. The tool demonstrates strong capabilities in conducting ANOVA and generating violin plots for both simple and complex datasets, making it valuable for a variety of applications. It also works reliably with datasets from vastly different cultures, including bread wheat, durum wheat, and triticale, sugar beet, cocklebur and lettuce, corn and sunflower, and soybean. However, to maximize its potential, future research should prioritize enhancing its usability and exploring integration possibilities with other tools, thereby strengthening its role in plant phenotyping workflows.

Three-dimensional visualization techniques can be classified into active and passive categories. Active techniques utilize a controlled source of structured energy emission, such as a scanning laser or projected light pattern, in conjunction with a detector, such as a camera, to generate an image. Passive techniques rely on ambient lighting to form an image (Harandi et al., 2023). Point sets can contain noise originating from various sources, whether the point cloud was actively or passively generated. Generated point clouds often suffer from limited sensor accuracy and measurement errors caused by environmental factors. Therefore, it is crucial to promptly identify and eliminate these outliers to prevent their impact on the accuracy of the results. In addition to environmental factors, technical errors caused by human interference can also lead to inaccuracies in generated point clouds. Common errors may also include improperly calibrated equipment, misaligned sensors, or incorrect parameter settings during the data acquisition process. Furthermore, other plausible reasons for errors in point clouds could be occlusions, reflections, and varying surface properties of objects being scanned.

The results demonstrate that our framework is robust and suitable for diagnostics throughout the experiment, requiring no formal statistical knowledge or advanced tool expertise.

We are also exploring ways to make our tool more general and less reliant on TraitFinder. Aside from the naming conventions in the experiment.csv file, we have largely achieved this goal. An alternative to our current approach with data acquisition would be to utilize measurements from deep learning models. Recently, there has been a significant increase in the use of deep learning models for analyzing phenotypic data. This trend has become increasingly important for the advancement of plant phenotyping research, as the available phenotyping platforms can be broadly categorized into two types: previously discussed commercially available solutions that offer, among other features, data processing tools, which are often proprietary; and more affordable options based on RGB or multispectral cameras and LiDARs, typically implemented on self-built platforms for indoor or outdoor use, or on unmanned aerial vehicles (UAVs) (Gano et al., 2021; Gano et al., 2024). In this latter case, the processing tools must be developed independently, and deep learning models represent the most flexible option for this purpose. We plan to use StatFaRmer in tandem with open-source deep learning point cloud processing tools to reduce reliance on proprietary instruments and extract new biological features from existing point clouds. This process could benefit from a “sanity check” through parallel processing with previously explored features.

As previously mentioned, deep learning models represent a versatile option for the open-source analysis of phenotypic data. For example in a recent study, the researchers developed a high-throughput phenotyping method utilizing RGB and infrared time-series data obtained from unmanned aerial vehicles (UAVs) and a multi-modal image segmentation model in order to monitor and quantitatively assess the growth of soybean canopy (Yu et al., 2024). The study found that the RIFSeg-Net, a novel multimodal image segmentation model, outperformed traditional deep learning-based image segmentation networks in accurately extracting canopy cover from unmanned aerial vehicle (UAV) images. The study demonstrates the potential of high-throughput phenotyping to rapidly identify crop germplasm with favorable traits such as high yield, disease resistance, and improved quality. This method can assist breeders in developing novel varieties with increased productivity and resilience, thereby enhancing crop quality and yield simultaneously.

Another study proposes a method for automatically acquiring detailed traits of rice panicles based on time-series images, using the YOLO v5 and ResNet50 models, as well as the DeepSORT algorithm, to analyze the effect of nitrogen on panicle development during the heading and flowering stages (Zhou et al., 2023). The proposed approach achieved high accuracy in counting panicles (R^2^ = 0.96, root mean square error (RMSE) = 1.73), as well as in estimating the heading date (absolute error of 0.25 days). The study revealed that higher nitrogen application leads to an earlier initiation and longer duration of flowering, and a longer total duration from the beginning of vigorous flowering to the end of the process. This proposed technique provides a novel approach to analysis for agricultural experts, and the impact of nitrogen on rice heading and blooming may assist us in avoiding extreme weather conditions and achieving sustainable and stable food production.

Another research paper explores the use of Terrestrial Laser Scanning (TLS) to study seasonal and circadian rhythms in plants and leaves under standard and cold stress conditions (Jin et al., 2021). The methods used in the research paper involved the collection of LiDAR data along with environmental data such as photosynthetically active radiation (PAR), temperature, and relative humidity throughout the growing season. Seasonal rhythms in structural traits like azimuth and Plant Leaf Area Index (PLA) were consistent between plant and leaf levels, while leaf-level rhythms were more diverse, such as changes in leaf inclination angle. Circadian rhythms of certain traits were found to be opposite under cold stress and standard conditions, with environmental factors showing stronger correlations with leaf trait rhythms under cold stress, especially air temperature. The study highlights the potential of using time-series TLS to study crop chronobiology in outdoor environments, aiding in understanding plant rhythms and survival strategies in response to environmental changes.

Many advanced deep learning data analysis methods under development could greatly benefit from a reliable and transparent validation tool, enabling comprehensive evaluation of outcomes through straightforward, interpretable metrics. Conversely, StatFaRmer would enhance its effectiveness by collaborating with emerging platforms designed for measuring traits critical to breeding. This integration would help alleviate phenotypic measurements as a bottleneck in Genome-Wide Association Studies (GWAS), streamlining the research process and improving the accuracy of trait assessments.

In the realm of time series analysis, a work by (Han et al., 2019) delves into the nuances of exploring data dynamic patterns, notably through the application of fuzzy clustering analysis. The approach of the study, if implemented in the newer versions of StatFaRmer, would allow detecting new phenotypic traits hidden in the temporal profiles.

Moreover, the limitations of traditional ANOVA in time series analysis have motivated researchers to explore more sophisticated approaches (Spyroglou et al., 2021). introduced a novel methodology that integrates generalized linear mixed models with classical time series models, modernizing the analysis of longitudinal datasets in plant sciences.

Furthermore, the landscape of crop-specific point cloud segmentation tools has seen significant advancements, exemplified by the pioneering work (Li et al., 2023). By harnessing these state-of-the-art tools, researchers can now extract valuable traits with unprecedented accuracy, underscoring the urgency of modularizing StatFaRmer for broader accessibility beyond one specific platform.

These articles inspire us by clearly indicating pathways for the future improvement of StatFaRmer.

## 5 Conclusion

StatFaRmer is an open-source tool created as a Shiny dashboard that is useful for the analysis of time series datasets in CSV format with capabilities of outlier filtration, grouping based on multiple parameters simultaneously, and more advanced statistical methods for assessing the significance of effects. It can be easily copied and used through a web interface by any number of users.

In this series of studies, StatFaRmer has become essential to evaluate the outcomes obtained from digital time-series phenotyping due to its flexibility and a wide range of customizable parameters for analysis. During our work with this tool, we were able to explore a diverse range of plant cultivars and identify the factors that influence the condition of specific plants. For instance, in our latest experiment, we grew plants of various varieties, and because of this tool, it was possible to not only compare the growth patterns between different varieties but also assess the impact of various alleles by grouping the varieties based on these allelic variants.

## Data Availability

The datasets presented in this study can be found in online repositories. The names of the repository/repositories and accession number(s) can be found in the article/supplementary material.
